# The role of flower consumption in Howler Monkey Females’ diet: adjustment across reproductive states

**DOI:** 10.7717/peerj.20659

**Published:** 2026-01-30

**Authors:** Anna Gisbrecht, John F. Aristizabal, Juan Francisco Rodríguez-Landa, Laura Teresa Hernández-Salazar

**Affiliations:** 1 Laboratorio de Biología de la Conducta, Instituto de Neuroetología, Xalapa-Enríquez, Veracruz, Mexico; 2 Departamento de Ciencias Químico Biológicas, Universidad Autónoma de Ciudad Juárez, Mexico; 3 Laboratorio de Neurofarmacología, Instituto de Neuroetología, Universidad Veracruzana, Xalapa-Enríquez, Veracruz, Mexico

**Keywords:** Feeding behavior, Lactation, Tannins and flavonoids

## Abstract

**Background:**

Although the genus *Alouatta* is considered a folivore and frugivore species, the consumption of vegetative parts like flowers often represents a considerable percentage of their diet. Flowers are high in tannins and flavonoids, which are beneficial for the animals due to the antioxidant and anti-inflammatory properties. However, there is a notable lack of knowledge regarding the role of flowers in howler’s diet, especially for the specific needs of females. The objective of this study is to gain further insight into the role of flowers for howler monkey females’ diets during lactation.

**Methods:**

Between 2021 and 2023, we collected data on the feeding behavior of 20 wild *Alouatta palliata mexicana* females during non-lactation, early lactation and late lactation on the Agaltepec Island, Mexico.

**Results:**

In 670 contact hours, a total of 1,471 feeding sessions were recorded. From 29 documented feeding items, five were flowers from the trees of *Andira galeottiana*, *Bursera simaruba*, *Dendropanax arboreus*, *Gliricidia sepium* and* Spondias mombin*. Documented feeding items were collected and evaluated for their tannin and flavonoid content. The annual weight-based flower consumption was 12.5%, which increased to 30.3% during blooming months. The tannin content of the flower species exhibited considerable variation, with levels ranging from 0 to 2,038.0 µg/g. In contrast, the qualitative evaluation of principal groups of flavonoid content detected a pronounced presence of flavanones in all five species and flavones in three. Notably, early lactating females exhibited a higher consumption of flowers compared to non-lactating and late lactating females. Conversely, the floral tannin intake was the highest in late lactating and lowest in early lactating females. Our findings suggest that dietary choices of howler monkey females are influenced by seasonal flower availability, with polyphenols playing an important role, suggesting physiological and behavioral adaptations in response to reproductive demands.

## Introduction

The genus *Alouatta* is among the most extensively studied primates, with a particular emphasis on their dietary ecology. Once considered primarily folivorous (Milton, 1981), howler monkeys are now known to consume fruits and leaves in roughly equal proportions (Estrada, 1984; Galetti, Pedroni & Morellato, 1994), sparking ongoing debate about their true level of frugivory (Giraldo et al., 2007; Dias & Rangel-Negrín, 2015; Garber, Righini & Kowalewski, 2015; Raño et al., 2016; Aristizabal et al., 2016). Despite the extensive research into the diet of these primates, relatively little attention has been given to female-specific nutritional needs, such that are related to reproductive physiology or health-related dietary adaptations. Given the hormonal and physiological fluctuations that female mammals experience throughout adulthood, and the close link between nutrition and reproductive success (McEvoy & Robinson, 2002; Kudesia et al., 2021; Vitikainen et al., 2023) it becomes especially plausible to assess the significance of particular food items within this context. Further, understanding the nutritional value of a food item for an animal’s diet benefits from examining its role across different physiological contexts. For example, evaluating food consumption in adult females throughout their reproductive cycle could provide valuable insight into the items’ functional importance (McEvoy & Robinson, 2002; Fontaine, 2012; Torre et al., 2014; Koga et al., 2020; Wohlgemuth et al., 2021; Kudesia et al., 2021; Skoracka et al., 2021; Vitikainen et al., 2023).

An often overlooked but ecologically relevant component in the diets of howler monkeys are flowers. Multiple studies note flower consumption (Silver et al., 1998; Cristóbal-Azkarate & Arroyo-Rodríguez, 2007; Dunn, Cristóbal-Azkarate & Veà, 2010; Fernández, 2014) with some even reporting patch depletion upon consumption (Righini et al., 2020), but do not address further implications. The broad diversity of flowers species mentioned in different studies which are consumed when seasonally available (Silver et al., 1998; Estrada et al., 1999; Cristóbal-Azkarate & Arroyo-Rodríguez, 2007; Chaves et al., 2023) further indicates that flowers may serve a greater role than acting as fallback foods in times of fruit scarcity (Lappan, 2009; Garber, Righini & Kowalewski, 2015). Furthermore, unlike other platyrrhines, howler monkeys possess trichromatic vision in both sexes, enabling them to discriminate flowers from the cryptic foliage background (Hernández-Salazar, Dominy & Laska, 2015). This ability strongly suggests that flower consumption is neither random nor accidental, but rather a selective and intentional feeding behavior. However, the nutritional significance of flowers for howler monkeys remains unknown. But flower consumption may hold special value for female howler monkeys, potentially contributing to their ability to meet varying nutritional demands during their reproductive cycle. Because different reproductive stages are accompanied by distinct metabolic and micronutrient requirements (Serio-Silva, Hernández-Salazar & Rico-Gray, 1999; Picciano, 2003; Power & Schulkin, 2006; Marshall et al., 2022), a stage-specific evaluation of flower use may help uncover functional dietary roles that would otherwise remain obscured in population-level dietary averages.

However, this evaluation can be challenging. Flowers make up a relatively small portion of howler monkeys’ overall annual diet, which might also be the reason for the limited research on flowers as a feeding source. In the literature that mentions flower consumption, annual portion of flowers ranged from 2.77% to 11% (Silver et al., 1998; Cristóbal-Azkarate & Arroyo-Rodríguez, 2007; Righini et al., 2020). Flowers are highly seasonal often appearing only during narrow temporal windows (Dunn, Cristóbal-Azkarate & Veà, 2010). However, these short bursts of availability may create ecological pressures that shape selective foraging strategies, that are likely especially acute for reproductive females with elevated demands. Evaluating flower consumption on a seasonal rather than annual basis may thus offer a more accurate understanding of its significance within howler monkey feeding ecology, particularly for females navigating distinct physiological stages. This raises the question of what factors might drive flower consumption despite their limited availability and overall dietary contribution.

One reason for howler monkeys to consume flowers could be the sugar abundant in the nectar and pollen (Fernandes et al., 2017). However, given their high-fiber diet, largely consisting of foliage, howlers have evolved hindgut fermentation (Amato & Righini, 2014; Garber, Righini & Kowalewski, 2015), a digestive system not well-suited for processing sugars (Stoner, Riba-Hernández & Lucas, 2005). Regardless, the individual components of flowers are too small to meet any significant nutritional needs (Lappan, 2009), suggesting that howlers consume flowers as a whole resource (Jones, 2005). A review of over 100 studies on edible flowers concluded that they are high in polyphenols like tannins and flavonoids (Fernandes et al., 2017). Thus, the potential benefits of flower consumption for howler monkeys may be due in part to their tannin and flavonoid content, among others. Tannins and flavonoids are both polyphenols, but they differ in their roles in plants and their physiological effects on animals. Tannins are considered to function as plant protection agents as they have a strong affinity to binding proteins, thereby reducing nutrient uptake and potentially damaging the digestive tract of animals (Sieniawska & Baj, 2017). There are two types of dietary tannins that differ greatly in structure: condensed tannins and hydrolyzed tannins (Sieniawska & Baj, 2017). As for flavonoids, they are important plant pigments that are widely distributed in flowers and fruits and protect the plant cell from oxidative stress and UV damage (Jäger & Saaby, 2011). Flavonoids are generally classified into seven subgroups (flavonols, flavones, isoflavones, anthocyanidins, flavanones, flavanols, and chalcones) (Jäger & Saaby, 2011). The consumption of flavonoids is beneficial for the animal as these compounds have strong antioxidant and anti-inflammatory properties and trigger various cellular processes enhancing cellular health and therefore general and mental well-being (Gutierres et al., 2014; Winter & Bickford, 2019; Cueto-Escobedo et al., 2020).

The connection to mental well-being could be particularly relevant for females during the postpartum period, when emotional sensitivity is elevated. Primate females undergo supraphysiological hormonal fluctuations throughout their reproductive cycle (Beehner et al., 2006; Power & Schulkin, 2006; Weltring et al., 2012; Rangel-Negrín et al., 2014; Hall & Klein, 2017) which are most prominent peri-partum (Hendrick, Altshuler & Suri, 1998; Power & Schulkin, 2006). The levels of steroid hormones progesterone and estrogen decline significantly following parturition (Hendrick, Altshuler & Suri, 1998). This abrupt decline in hormone levels is frequently accompanied by emotional instability in the mother, and may result in postpartum depression since progesterone and estrogen show anxiolytic- and anti-depressant-like properties in women and experimental female animals (Hendrick, Altshuler & Suri, 1998; Rupprecht, 2003; Zuluaga et al., 2005) much like flavonoids (Colombage et al., 2024). In this context, the consumption of flavonoid-rich flowers could contribute to stress regulation and overall fitness (Rodríguez-Landa et al., 2022; German-Ponciano et al., 2022).

Contrariwise, lactating females are more likely to be negatively affected by protein precipitation properties of tannins. The lactation phase is characterized by an increased protein and fat requirement (Serio-Silva, Hernández-Salazar & Rico-Gray, 1999; Houdijk, Jessop & Kyriazakis, 2001). Given that tannins precipitate proteins in the diet, it would be advantageous for lactating females to reduce their dietary tannin intake during lactation to utilize the available proteins for milk production (Sieniawska & Baj, 2017). Interestingly, howler monkeys have adapted to a tannin-rich diet by binding tannins in the oral cavity with proline-rich proteins, which are abundant in their saliva (Espinosa-Gómez et al., 2018). Therefore, the feeding behavior of the females in any reproductive state should not be affected by the tannin content of the flowers.

Given the potential benefits of flavonoids for emotional regulation and howler monkeys’ adaptation to tannin-rich diets through proline-rich salivary proteins, we propose the following hypotheses. First, due to the hormonal changes and emotional imbalance following parturition (Hendrick, Altshuler & Suri, 1998; Beehner et al., 2006; Weltring et al., 2012; Rangel-Negrín et al., 2014), we predict that lactating females will increase their flower consumption—and consequently, their intake of flavonoids—during the early stages of lactation. Second, although flowers often have high levels of tannins (in addition to flavonoids), we expect that the tannin content of flowers will not strongly influence the diet of lactating females due to their physiological adaptation. Therefore, we predict no significant difference in the consumption of floral *versus* foliar tannins across reproductive states, but an increase in floral (flavonoid-rich) consumption during lactation.

## Methods

### Ethics

This study is noninvasive and adheres to the American Society of Primatologist (ASP) Code of Best Practices in Field Primatology and the ASP Principles for the Ethical Treatment of Nonhuman Primates. Approval of research protocols from the Secretaría de Medio Ambiente y Recursos Naturales. The research complies with the legal requirements of Mexican law (NOM 059-SEMARNAT-2010; SEMARNAT SGPA/DGVS/04015/21).

### Field site

Field work was carried out on Agaltepec Island with an area of 8.3 ha (18°24′–18°25′N, 95°05′–95°06′W) located in Lake Catemaco, Veracruz, Los Tuxtlas, Mexico. The island includes flora of two vegetation types: semi-evergreen forest with several areas of secondary vegetation with 65 identified tree species corresponding to 32 families and 58 genera and a mean canopy height of 15–20 m. The region’s climate is warm-humid, with an annual rainfall of 1,980 mm. The average annual temperature is 23.4 °C ranging from a minimum of 17 °C to a maximum of 31.9 °C, with the dry season from March to May and the rainy season from June to February (Asensio et al., 2007; Rodríguez-Luna et al., 2003).

### Study animals and data collection

A group of wild and fully habituated mantled howler monkeys (*Alouatta palliata mexicana*) (23 adult females, 13 adult males and nine juveniles) (Gisbrecht, Hernández-Arriaga & Hernández-Salazar, 2023) was monitored between September 2021 and August 2023. The project focused exclusively on adult females. Further, due to identification constraints, only 20 adult females were included in his study. The females were identified by means of pelage coloration and certain physiological features (Gisbrecht, Hernández-Arriaga & Hernández-Salazar, 2023). The state of lactation was divided into two periods: “early lactating” (EL) and “late lactating” (LL). EL was determined through the presence of a dependent offspring (birth to three months) carried mostly ventrally by the mother (Balcells & Veà Baró, 2009; Cano-Huertes et al., 2017). Hereafter, the lactation state of the female was identified as “late lactating” (LL) where the offspring was increasingly independent from its mother and consumed solid food progressively (Balcells & Veà Baró, 2009; Cano-Huertes et al., 2017). These two lactating states were contrasted with the “non-lactating” stage (N/L). In the observation period we collected data on 17 N/L, six LL and five EL females. Due to the project’s length, some females could be observed throughout all three stages. This also allowed us to rule out gestation in N/L females.

Female behavior was collected every month for 10–20 days throughout the month, when possible (except for December and January), from sunrise and the following 5 h, during which the most feeding occurred, in a random order ensuring that all females were recorded per day. We recorded the time invested (seconds) and food intake (grams) of the items consumed by the females following the categories: mature leaves (ML), young leaves (YL), mature fruits (MF), immature fruits (IF), flowers (FL), and petioles (PT). To calculate a food intake rate, we recorded information on the number of discrete food units (*i.e.,* individual items or batches of items in 1-minute bite-rate) consumed during a feeding session. This rate was multiplied by the time of simultaneous feeding of the whole group which allowed the calculation of grams ingested for a feeding bout and then the total tannin intake by females on a dry weight-basis (grams) (Aristizabal et al., 2016).

\begin{eqnarray*}GF& = \left[ g \right] fs\times \left[ min \right] gf \end{eqnarray*}

\begin{eqnarray*}TNgf& = \left[ g \right] fs\times TN \end{eqnarray*}

where: GF—grams ingested during simultaneous group feeding, [g]fs—grams of food source ingested in feeding session, [min]gf—time of simultaneous group feeding in minutes, TNgf—tannins ingested during simultaneous group feeding and TN—the proportion of tannins in one gram of the food source.

To measure the mass of food, we first collected all consumed and dropped fruits or fruits in the same condition as consumed from that tree. Leaf and flower samples were collected from the feeding-tree in the condition most similar to consumed items. Subsequently, leaves were dried in a cardboard box using the heat of a 60 W light bulb, and fruits and flowers were dried in a food dehydrator set at 35 °C. The samples were evaluated for flavonoid content at the Instituto de Neuroetología, Universidad Veracruzana, and tannin content at the Instituto de Química Aplicada, Universidad Veracruzana.

### Preliminary flavonoid content determination

The presumptive presence of flavonoids was determined by calorimetry methods. The extracts of flowers were subjected to phytochemical analysis using preliminary quantitative methods through standardized techniques to detect the presence of secondary metabolites groups (Domínguez, 1973; Cseke et al., 2006), in this case flavonoids as flavones, flavanones, flavanols, flavanonols, xanthones, aurores, leucocyanidins, chalcones and catechins (Rodríguez-Landa, Hernández-Lozano & Méndez-Ventura, 2020). A quantity of 0.5 g respectively of dried and pulverized leaf, flower or fruit samples was mixed with five ml of methanol, ethanol (96%), chloroform or ether resulting in four mixtures. To obtain the extracts, each of the four mixtures was heated to a gentle simmer and left to simmer for 5 min. After a cooling phase, the extracts were filtrated from residual plant particulates. Each extract was subjected to the following reaction: three drops of extract, a piece of magnesium in metallic form and four drops of HCL; three drops of extract and three drops of H_2_SO_4_; three drops of extract, a scrap of pulverized zinc and four drops of HCL, respectively. The occurrence of specific colorations indicates the presence of certain types of flavonoids.

### Tannin content determination

The dried and pulverized samples were processed into solvents with methanol HPLC grade at a ratio of 1:3. The methanol/powder mixture was vortexed for one minute, let sit for five minutes and then passed through a 0.045 µm pore size nylon filter to separate the extract from the residual mixture. To determinate the tannin content in the extract, the High-Performance Liquid Chromatography with Variable Wavelength Detection (HPLC-VWD) method was applied to separate, identify and quantify the components of a mixture. For our analysis, the HPLC equipment Agilent Technologies, model 1,200 infinity with a UV–VIS detector set at 270 nm was used. A 3.5 mM octadecylsilane (C18) column (4.6 × 100 mm) was used to separate the tannins in the extract. As an injector for the mobile phase A (acetonitrile), B (methanol), and C (water) were applied (15% A; 15% B; 70% C) at a 0.5 mL/min flow rate and with a five µL injection volume, at a temperature of 20 °C.

### Statistics

First, the consumption of fruits (FR), flowers (FL) and leaves (LF) quantified for each individual female, in each physiological state, based on the grams was consumed over a standardized 10-day observation period. Physiological states were coded as a three-level factor: non-lactating (N/L), early lactating (EL), and late lactating (LL). To evaluate the effects of the predictor variables (month, reproductive state, lactation state, food item—flowers *vs.* leaves) on the food intake (grams consumed) and tannin intake (grams consumed) we used generalized linear mixed model (GLMM 1 and GLMM 2, respectively; see Table 1). The models were fitted using the Gamma family with an inverse link, which is appropriate for continuous and positive data with a variance that increases with the value of the mean (Cayuela Delgado & De la Cruz, 2022). A random effect for Animal ID was included to account for repeated measures within individuals. We initially included all biologically plausible two-way interactions in the full models and used a backward stepwise approach to remove non-significant interactions, retaining only significant terms in the final models. If in GLMM 2 no significant interactions were detected, we re-ran the model removing those terms and retained only their main effects. We use a likelihood ratio test to compare each full model (model with predictor variables) to a corresponding null model that included only the intercept (Dobson & Barnett, 2018) (Table 1). We did not detect any issues of non-convergence, overdispersion, or under dispersion in any of our models. All analyses were performed in R software version 4.3.2 (R Core Team, 2024), with the lme4 package for model fitting (Bates et al., 2015).

**Table 1:** Hypotheses tested, full models, and results of the likelihood (Saturated model *vs* simplified model) ratio test for each model. In GLMM 2 the “*” represents the interactions between variable levels.

Hypothesis tested	Full model	Likelihood ratio test
Food intake correlates with month, physiological state and item	GLMM 1: food intake (gr) ∼ Month + Physiological state (lactating, non-lactating) + Lactating state (non-lactating, early lactating, late lactating) + food type (Flower, Leaf)	*χ*^2^ = 14.5, *df* = 3, *P* < 0.01
Tannin intake correlates with month, physiological state and item	GLMM 2: tannin intake (gr) ∼ Month + Physiological state (lactating, non-lactating) + Lactating state (non-lactating, early lactating, late lactating) + food type (Flower, Leaf) + Lactating state * food type	*χ*^2^ = 53.1, *df* = 4, *P* < .001

## Results

### General flower consumption

**Figure 1:**
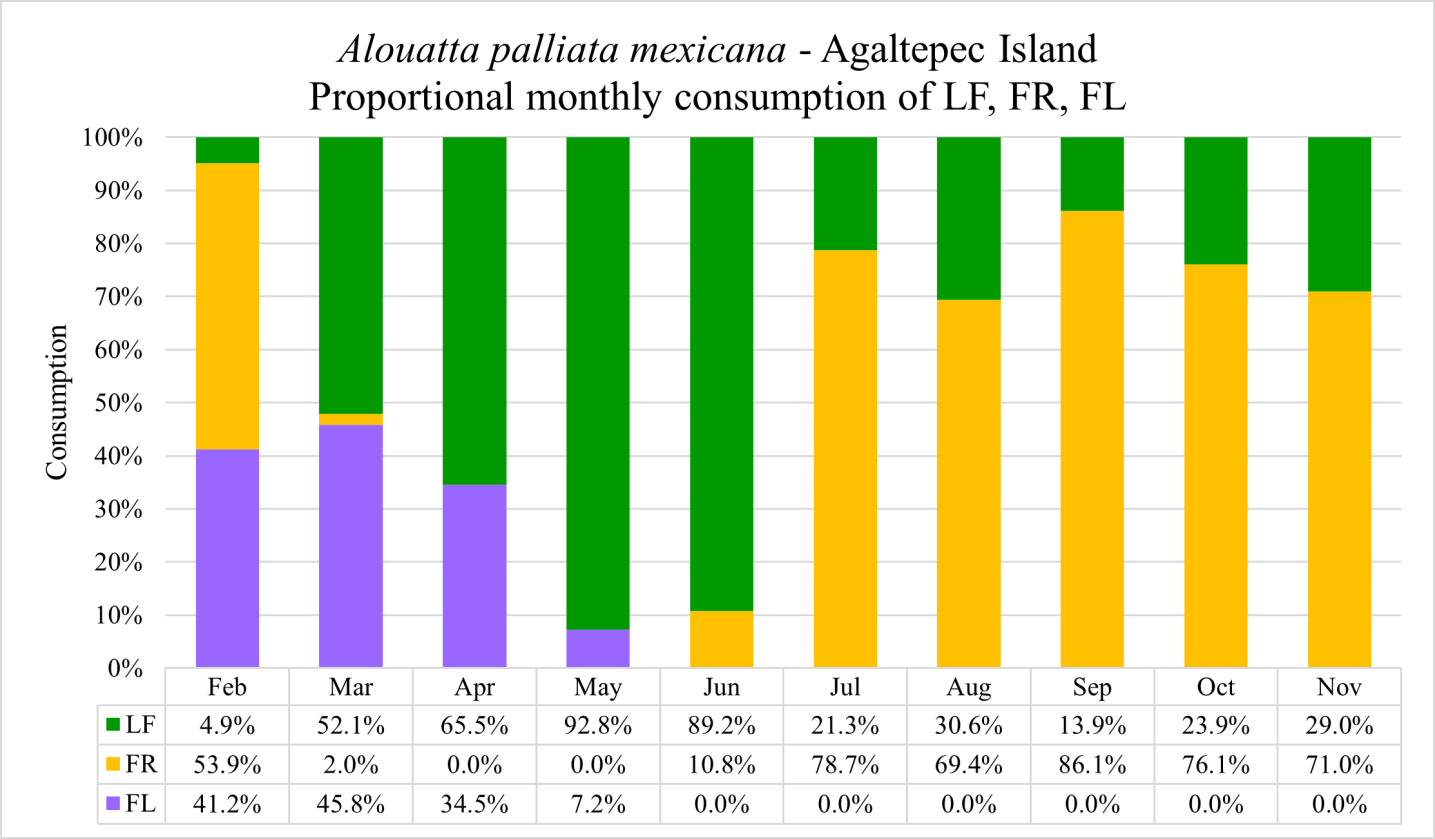
Monthly proportions of flowers (FL), fruits (FR) and leaves (LF) consumption by *Alouatta palliata mexicana* on the Agaltepec Island between 09.2021–08.2023. One-minute bite rates of discrete food units were multiplied by the time of simultaneous feeding of the entire group. Using the data on grams of leaves, fruits and flowers consumed within a month, we calculated the percentage of consumption of these feeding items.

During the field period 09.2021–08.2023, a total of 1,471 sessions with 670 contact hours were recorded (see data in repository: Gisbrecht et al., 2024). The group exhibited a consistent consumption pattern where the general consumption of the females is indicative for the entire group. The monkeys fed on 13 different plant species and five different item categories (Table 2). The annual portion of leaves, fruits and flowers intake was 45.0%, 42.5% and 12.5%, respectively. The highest monthly intake of flowers was observed in March (45.8%) followed by February (41.2%), April (34.5%) and May (7.2%) (Fig. 1). However, when the percentage of intake per month is considered in the context of annual consumption, April exhibits the highest flower intake (6.0%), followed by March (4.8%), February (1.0%) and May (0.8%). Howler monkeys fed on flowers of *Gliricidia sepium* (34.9%)*, Spondias mombin* (25.2%), *Dendropanax arboreus* (23.1%), *Bursera simaruba* (11.0%), and *Andira galeottiana* (5.7%). In the case of *G*. *sepium* and *B*. *simaruba* the feeding occurred until patch depletion. When analyzing the data, we did not find significant differences (*U* = 4,057, *p* > 0.05) between the consumption (grams) of flowers (mean ± SD; 119.9 ± 81.5 g) and leaves (153.1 ± 118.4 g) during the flowering season (Feb–May). Meanwhile, we found significant differences (*U* = 2,943, *p* < 0.001) in tannin intake between flowers (mean ± SD; 0.042 ± 0.009 g) and leaves (0.006 ± 0.001 g). Likewise, we found significant differences in flower intake (grams) among non-lactating (*n* = 17), early lactating (*n* = 5), and late lactating (*n* = 6) females (ANOVA: *F* = 7.17, *df* = 3, *p* < 0.01), with early lactating females consuming significantly more flowers than non-lactating females (Tukey pair test, *p* < 0.01).

**Table 2:** Species, families and the plant-part consumed by *Alouatta paliatta mexicana* females. Mature leaves (ML), young leaves (YL), mature fruits (MF), immature fruits (IF), flowers (FL), petioles (PT).

Species	Family	Parts consumed
*Andira galeottiana*	Fabaceae	IF, ML, YL, FL, PT
*Bursera simaruba*	Burseraceae	IF, ML, FL
*Dendropanax arboreus*	Araliaceae	FL, IF
*Ficus cotinifolia*	Moraceae	IF, YL, PT
*Ficus lundellii*	Moraceae	ML, PT
*Ficus perforata*	Moraceae	ML
*Gliricidia sepium*	Fabaceae	FL, ML, YL
*Ipomea batatas*	Convolvulaceae	ML
*Lonchocarpus cruentus*	Fabaceae	ML
*Philodendron tripartitum*	Araceae	PT
*Protium copal*	Burseraceae	MF, IF
*Sideroxylon capiri*	Sapotaceae	MF, IF
*Spondias mombin*	Anacardiaceae	MF, ML, YL, FL

### Flavonoid and tannin content determination

All flower samples demonstrated a strong reaction for the presence of flavanones which is a subgroup of flavonoids. Additionally, flowers of *B. simaruba, S. mombin* and *G. sepium* tested positively for the presence of another subgroup, flavones. There was very little to no evidence for the remaining flavonoid groups tested (flavanols, flavanonols, xanthones, aurores, leucocyanidins, chalcones, catechins). The HPLC-VWD analysis for tannins revealed higher values in flowers of *B. simaruba* (2,038.0 µg/g) and *A. galeottiana* (1,117.1 µg/g) compared to *G. sepium* (190.9 µg/g), whereas *D. arboreus* and *S. mombin* tested negative to tannin presence (Table 3).

### Effects of female states on food and tannin intake

**Figure 2:**
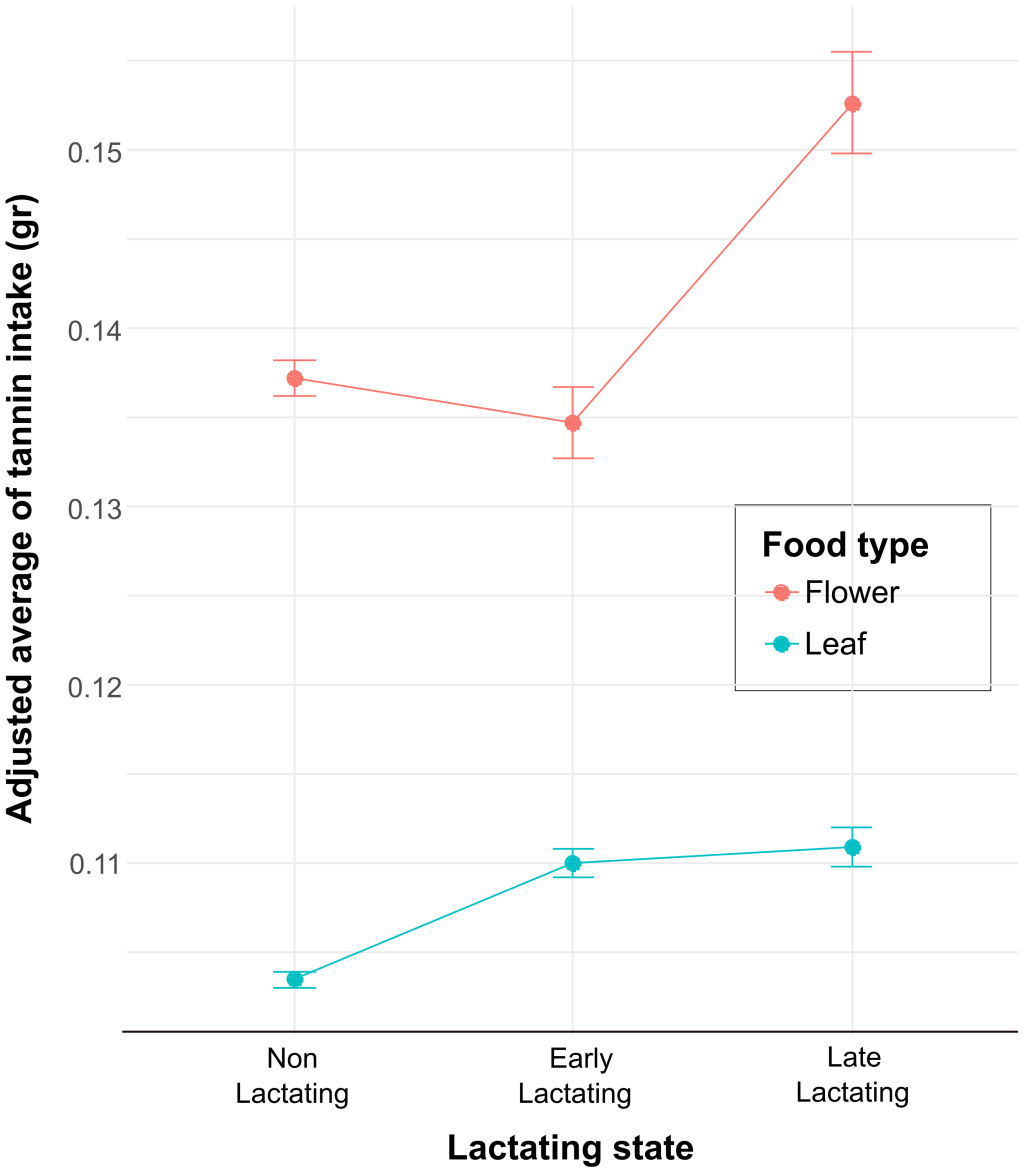
The interaction plot shows the adjusted means of the tannin intake as a function of the levels of the categorical variable lactating state for the two levels of the food type variable (FL and LF). The values on the Y axis represent the adjusted means of tannin intake (points) and the standard error (95% confidence intervals) predicted by the model fitting and reflect the average estimates of tannin intake for each combination of food type and lactating state.

Each full model and the corresponding likelihood ratio test results are shown in Table 1. The first model (GLMM 1) showed that physiological state and food type influenced the quantity of food intake (Table 4). The non-lactating reproductive state shows a negative and significant effect on food intake (Estimate = 0.03, SE = 0.01, *t*-value = 3.63, *p* < 0.001), indicating that, on average, they consume 0.03 less units of food compared to the reference lactating level (Table 4). On the other hand, leaves as a food type have a positive and marginally significant effect on food intake (Estimate = −0.02, SE = 0.01, *t*-value = −1.95, *p* < 0.05). This 0.02-unit increase suggests that leaves were consumed more compared to flowers. The second model (GLMM 2) showed that the interaction between the levels of the variables lactating state and food type influenced tannin intake (Table 4). The interaction between food type flower and non-lactating was significant (Estimate = −1.87, SE = 0.66, *p* = 0.004) compared with the reference level interaction (Leaf: Early lactating), indicating that this reference level interaction is associated with a decrease in tannin intake. Likewise, the interaction between flower and late lactating, and flower with early lactating was also significant (Table 4). Finally, we calculated the adjusted mean from the model estimations for the interactions in the GLMM2, and tannin intake from flowers shows a clear pattern of increase at the late lactating level and then a decrease at the early lactating level (Fig. 2). Whereas, for the food type LF, the adjusted mean of tannin intake remains relatively constant across levels, with a slight increase from N/L to LL and EL. On the other hand, across all reproductive states, the food type FL showed higher adjusted means of tannin intake compared to the food type LF. This indicates that FL is associated with higher tannin intake across all conditions of lactating state. Finally, the error bars (Fig. 2) represent the variability of the estimates by the model. For the food type FL, especially at the LL level, there is greater variability compared to the food type LF, which could indicate more heterogeneity in the measurements of tannin intake for food type FL in this condition.

**Table 3:** Flower species consumed by *Alouatta paliatta mexicana* females indicating presence of flavanones/ flavones and tannin content.

Flower species	Flavanones	Flavones	Tannins (µg/g)
*Andira galeottiana*	x		1,117.1
*Bursera simaruba*	x	x	2,038.0
*Dendropanax arboreus*	x		0
*Gliricidia sepium*	x	x	190.9
*Spondias mombin*	x	x	0

**Table 4:** Results of the models examining the effects of month, physiological state, lactation state and item on the food intake (GLMM 1) and tannin intake (GLMM 2) in mantled howler monkeys. *P*-values for t test of the significant variables. In brackets the reference levels of the significant variables. In GLMM 2 the “*” represents the interactions between variable levels. LF, leaves; FL, Flowers; L, Lactating; N/L, non-lactating; EL, early lactating; LL, late lactating.

Model		Estimate	SE	t-value	***P***
GLMM 1					
	Intercept **(L and FL)**	0.07	0.01	7.85	<0.001
	Physiological state ** (N/L)**	0.03	0.01	3.63	<0.001
	food type ** (LF)**	−0.02	0.01	−1.95	<0.05
GLMM 2					
	Intercept **(LF:EL)**	9.27	0.60	15.41	<0.001
	food type (FL)* Lact. State ** (N/L)**	−1.87	0.66	−2.85	<0.01
	food type (FL)* Lact. State ** (LL)**	−2.70	0.68	−3.99	<0.001
	food type (FL)* Lact. State ** (EL)**	−1.53	0.82	−1.85	<0.05

## Discussion

In this study, we showed that flowers appear to be a popular food source for howler monkeys when available. Fruit consumption was very scarce in the blooming season (*n* = 5), as almost no fruits were available during this time of the year on the Agaltepec Island. Therefore, consumption of fruits will be neglected in this discussion. Lactating females spent more time consuming food overall (186.9 ± 124.5 g, *n* = 77) compared to non-lactating females (120.60 ± 96.49 g, *n* = 136). Non-lactating females consumed less of all items, with leaves averaging at 131.94 ± 108.0 g (*n* = 89) and flowers at 97.38 ± 63.82 g (*n* = 46). Generally, in lactating females, leaves were also the most consumed item (190.02 ± 125.19 g, *n* = 51) followed by flowers (167.12 ± 91.82 g, *n* = 22). This demonstrates that, while there was a general increase in consumption, lactating females increased consumption of leaves only by 144% while consumption of flowers was increased by 171%, which is a significant difference and supports our prediction. Interestingly, between the lactating states (LL and EL) there were discrepancies in flower and leaf consumption in consideration of the quantities consumed. While LL females had a higher consumption of leaves (205.28 ± 129.80 g, *n* = 28) over flowers (153.98 ± 92.63 g, *n* = 15), EL females showed the opposite with flower consumption (195.27 ± 83.34 g, *n* = 7) exceeding leaf consumption (171.44 ± 116.67 g, *n* = 23). These results indicate that lactating females not only increase their overall intake but also consume relatively more flowers compared to non-lactating females. Further, it shows that flowers appear to play an important role in the diet of howler monkeys’ females during the early lactation-period. Further, we detected differences in tannin consumption between lactating and not lactating females, with tannin consumption being higher in lactating females (EL and LL combined). Ultimately, the evaluations showed differences in foliar and floral tannin consumption as well. The differences were unequally distributed along the stages of lactation, however, with late lactating females consuming significantly higher amounts of floral tannins than early lactating and non-lactating females.

The absence of significant differences in the consumption of leaves and flowers during the blooming season (Table 4, GLMM 1) suggests that flowers may play a more important role in the diet of howler monkeys’ females than as a fallback option (Asensio et al., 2007; Cristóbal-Azkarate & Arroyo-Rodríguez, 2007). Further, it indicates that regarding the role of flower as a percentage of consumption within the annual diet might be inaccurate, as flowers are not available throughout the year. The nutritional value of flowers for the diets of howler monkeys should be assessed seasonally.

Within the flower consumption and in consideration of the reproductive state, EL and LL females consumed significantly more flowers than N/L females, with LL showing the highest levels of consumption. The significant increase in consumption during early lactation was expected because EL females experience mental distress due to decreased progesterone and estrogen levels post-partum (Pepe & Albrecht, 1995; Hendrick, Altshuler & Suri, 1998; Beehner et al., 2006; Weltring et al., 2012; Rangel-Negrín et al., 2014; Hall & Klein, 2017; Ma et al., 2019; Pinna, Almeida & Davis, 2022; Etyemez et al., 2023). These hormones and their derivatives play a role in both gestational progression and mental health in general, acting as anxiolytics and antidepressants (Hendrick, Altshuler & Suri, 1998; Rupprecht, 2003; Zuluaga et al., 2005). Flavonoids are strong antioxidants decreasing oxidative stress and inflammation, which are known to contribute to the development of neurodegenerative and neuropsychiatric disorders (Rodríguez-Landa et al., 2022; German-Ponciano et al., 2022). Therefore, flavonoids are also anxiolytic and antidepressant agents operating in similar ways to progesterone and estrogen (Kiyama, 2023; Widiawati et al., 2024). Consequently, the females in our study may counteract the negative effects following the hormone decrease by consuming nature’s antidepressants: flavonoids (here specifically flavanones and flavones). It is a hypothesis that requires specific studies to be supported or discarded.

Following the elevated consumption of flowers by lactating females, we also found that they consumed more tannins from flowers than from leaves. This on itself is not surprising, some of the flower species tested for substantially higher tannin levels compared to leaves and fruits. Additionally, howler monkeys are able to disarm tannins in their food source by producing proline-rich proteins in their saliva, which bind tannins very effectively and enables the monkeys to consume food with high tannin content (Espinosa-Gómez et al., 2018). However, when comparing different stages of lactation, the data reveals a more intriguing pattern. Early lactating females ate more flowers than non-/late lactating, but their intake of tannins from flowers was about the same. In contrast, late lactating females consumed significantly more flowers and floral tannins with them (Fig. 2). This suggests that EL females selectively avoided flowers with high tannin content while still increasing their flower intake. LL females, on the other hand, seemed to seek out tannin-rich flowers.

These behaviors imply that tannins might play a physiological role for lactating females after or despite the neutralization by proline-rich proteins in their saliva. Their intake could serve functions which play a higher role than the elevated need for proteins, since tannins decrease protein-intake (Sieniawska & Baj, 2017). This possibility has already been observed in other animals. Grazing livestock animals have also developed an adaptation to precipitate tannins from their food through tannin binding proteins in their saliva (Ward, Schmitt & Shrader, 2020), similar to the adaptation of howler monkeys (Espinosa-Gómez et al., 2018). And yet, for ruminant females an increased milk yield is often reported due to a diet rich in condensed tannin and increased milk-quality after a diet rich in hydrolysable tannins (Tan et al., 2015; Davidović et al., 2019; Raza et al., 2022; Gannuscio et al., 2022; Prapaiwong et al., 2023).

This project took place in field conditions on wild animals; therefore, it is challenging to ascertain the physiological requirements underlying the selectivity of tannin consumption in howler monkeys’ females. The early lactation period is a critical phase during which the animal’s nutritional requirements are high, due to the need for recovery from parturition and the onset of milk production. The influence of a diet high in tannins during this period can be multifaceted and complex. Research on this topic is inconclusive, with studies reporting both benefits and disadvantages of a condensed tannin-rich diet in the early postpartum period (Kabasa et al., 2004; Sallam et al., 2019). There are also no conclusive studies on the influence of hydrolyzed tannins or molecular size of tannins in general on female or offspring physiology in the early postpartum period.

On the account of habitat destruction, howler monkeys face shrinking and fragmented habitats. They are also greatly affected by the illegal pet trade, which forces many animals to live in captivity, even after rescue (Pastor-Nieto, 2015). Howler monkeys are generalists but need to maintain a highly mixed diet to sustain fitness and population growth (Simpson & Raubenheimer, 2012). Their conservation and husbandry are challenging, as their diet composition is very complex to provide and frankly, not fully understood to date (Dáttilo et al., 2014; Amato et al., 2017; Silveira et al., 2024). Our research points to adjustment of reproductive females to the availability of flowers and the polyphenols coming with this food-item and therefore highlights that howler monkeys respond not only to internal, physiological needs (such as lactation), but also adapt their diet to external changes, such as seasonal food availability. Despite the valuable insights our study offers on the role of flower consumption in lactating *Alouatta palliata mexicana* females, we must acknowledge its limitations. The research was carried out over two years and focused on a single group of monkeys. While this provides a solid foundation, it may not fully reflect long-term trends or variations in the species’ feeding behavior. Longer-term studies are necessary to understand how dietary patterns shift in relation to reproductive stages. Moreover, the study’s time frame was too short to explore whether social factors such as relationships between individuals or group hierarchies influence females’ feeding behavior. Further and more comprehensive research is needed to investigate the dietary plasticity and its particularities in this species.

## Conclusion

This study reveals that flower consumption in *Alouatta palliata mexicana* is a strategic dietary choice. Howler monkeys increase the consumption of flowers during blooming months. Floral tannins appear to be important in the diet for females; and particularly, late-lactating females seek for high tannin content in the food. These behaviors likely reflect adaptations to fluctuating nutritional and psychological needs postpartum. It may not only contribute to the composition of food intake, but also to maintaining the health and well-being of lactating female monkeys, probably by reducing the stress associated with the lactation period. However, further research with an extended observation period is necessary to reach a more conclusive statement. In particular, studies of the effect of tannin rich diets in non-human primates and its relationship to milk quality and production. The hormonal decline after parturition and the possible self-medication by consuming natural antidepressants, flavonoids, should also be addressed.

## Additional Information and Declarations

### Field Study Permissions

The following information was supplied relating to field study approvals (*i.e.*, approving body and any reference numbers): The Secretaría de Medio Ambiente y Recursos Naturales.

### Data Availability

The following information was supplied regarding data availability: The data are available at figshare: Gisbrecht, Anna; Aristizabal, John F; Susana Hernández-Arriaga, Erandi; Rodríguez-Landa, Juan Francisco; Mendoza-Lopez, María Remedios; Hernández-Salazar, Laura T. (2024). The Role of Flower Consumption in Female Howler Monkeys: Adjusting Diet across Reproductive Stages. figshare. Dataset. https://doi.org/10.6084/m9.figshare.27902628.v2. The code is available at figshare: Aristizabal, John F; Gisbrecht, Anna; Hernández-Salazar, Laura T. (2024). Code files for: The Role of Flower Consumption in Female Howler Monkeys: Adjusting Diet across Reproductive Stages. figshare. Software. https://doi.org/10.6084/m9.figshare.27905409.v1.
